# Pediatric Meningoencephalitis Cluster Caused by Snowshoe Hare Virus, Whistler, British Columbia, Canada, 2024

**DOI:** 10.3201/eid3204.251392

**Published:** 2026-04

**Authors:** Faisal Ali, Miguel Imperial, Muhammad Morshed, David M. Goldfarb, Jonathan B. Gubbay, Catherine A. Hogan, Rohit Vijh, Ellie N. Andres, Marta Jaeckel, Jaskiran Sajan, Heidi Wood, Alyssia Robinson, Jennifer Tam

**Affiliations:** University of British Columbia, Vancouver, British Columbia, Canada (F. Ali, M. Imperial, M. Morshed, D.M. Goldfarb, J.B. Gubbay, C.A. Hogan, R. Vijh, J. Tam); Children’s & Women’s Health Centre of British Columbia, Vancouver (F. Ali, M. Imperial, D.M. Goldfarb, J.B. Gubbay, J. Tam); British Columbia Centre for Disease Control, Vancouver (M. Morshed, C.A. Hogan); Vancouver Coastal Health Public Health Surveillance Unit, Vancouver (R. Vijh, E.N. Andres, M. Jaeckel, J. Sajan); National Microbiology Laboratory, Public Health Agency of Canada, Winnipeg, Manitoba, Canada (H. Wood, A. Robinson)

**Keywords:** Snowshoe hare virus, encephalitis virus, arbovirus, arthropodborne viruses, viruses, vector-borne infections, zoonoses, meningitis/encephalitis, mosquitoes, encephalomeningitis, meningoencephalitides, pediatrics, California, British Columbia, Canada

## Abstract

Snowshoe hare virus (SSHV) is an arbovirus in the California serogroup known to circulate throughout Canada and northern latitudes of the United States. The clinical spectrum of SSHV infection ranges from asymptomatic or mild febrile illness to neuroinvasive disease; neuroinvasive disease occurs more often in children and young adults. We describe a cluster of confirmed and probable SSHV meningoencephalitis cases in 3 children from Whistler, British Columbia, Canada, in the summer of 2024. We highlight the shared epidemiology, clinical manifestations, serologic diagnostic methods, and outcomes for the cases. All 3 children acquired the infection locally and made a full recovery. This case series suggests underrecognized SSHV infection prevalence that warrants enhanced surveillance and review of existing diagnostic algorithms. California serogroup viruses, including SSHV, should be recognized as a potential cause of neuroinvasive disease in North America during mosquito season, particularly when initial diagnostic testing is inconclusive.

Snowshoe hare virus (SSHV) is an arthropodborne California serogroup virus (CSGV) of the genus *Orthobunyavirus* (*1*,*2*). SSHV was first isolated from a snowshoe hare (*Lepus americanus*) in Montana, USA, in 1958 and is primarily found across Canada and the northern latitudes of the United States (*2*). The virus is maintained in an enzootic cycle between small mammals, particularly snowshoe hares but also rabbits (*Oryctolagus* and *Sylvilagus* spp.), squirrels (*Sciurus* spp.), chipmunks (*Tamias* and *Neotamias* spp.), and other rodents, and mosquito vectors, primarily *Aedes* and *Culiseta* spp. mosquitoes (*1*,*2*). SSHV transmission typically occurs during mosquito season (May–September in North America) through blood meal bites (*3*), and the incubation period is ≈3–7 days (*2*).

Most SSHV infections in humans are asymptomatic or cause mild illness, but the virus can lead to severe neuroinvasive disease, especially in children and young adults. Symptoms of SSHV infection can range from febrile illness to meningoencephalitis (*2*). Despite its potential for high rates of illness, SSHV infections are likely underdiagnosed and underreported.

SSHV infection diagnosis is challenging because clinical manifestations are nonspecific and often mimic those of other viral infections. The National Microbiology Laboratory in Canada offers molecular and serologic testing for SSHV and Jamestown Canyon virus (JCV), the 2 most common CSGVs that are known to circulate in Canada. Although reverse transcription PCR (RT-PCR) can differentiate between the CSGVs, it has lower sensitivity because of the low and transient viremia in immunocompetent persons, particularly at the stage of illness when they tend to be hospitalized. Serology is more sensitive, but IgM to CSGVs like SSHV and JCV cross-react when tested by ELISA. Most case definitions for a confirmed case of CSGV incorporate the comparison of acute and convalescent serum neutralizing antibody titers by a plaque reduction neutralizing assay (PRNT), generally requiring a change of >4-fold (*2*,*4*–*6*). We describe a cluster of 3 pediatric meningoencephalitis cases caused by SSHV infection in Whistler, British Columbia, Canada, during July–August 2024. 

## Cases

In summer 2024, three pediatric patients were admitted to British Columbia’s pediatric tertiary hospital with cerebrospinal fluid (CSF) culture-negative meningoencephalitis. The 3 patients had similar clinical manifestations, lived in the same geographic area (Whistler, BC, Canada), had symptom onset dates within 1 week of each other (Figure), and had negative initial testing on first-tier and targeted infectious diseases workups. 

**Figure:**
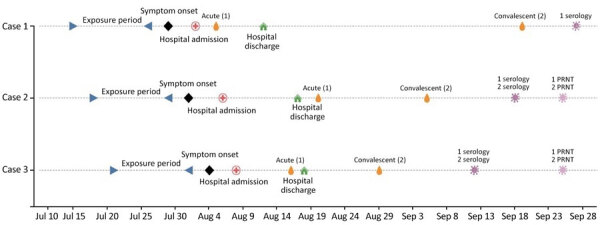
Chronological timeline of pediatric meningoencephalitis cases caused by snowshoe hare virus, Whistler, British Columbia, Canada, 2024. PRNT, plaque reduction neutralization test.

When the treating clinicians recognized an epidemiologic link among the 3 cases, they alerted public health and expanded the diagnostic approach after patients clinically improved and were discharged. We obtained written consent to describe cases from all 3 patients’ guardians.

### Case 1

In late July 2024, a 12-year-old girl with no underlying conditions was seen in the emergency department with a 3-day history of severe, bilateral, pulsatile headache and associated photophobia and phonophobia and a 1-day history of abdominal pain and emesis. She was presumed to have a migraine and discharged home with regular analgesia.

The next day, day 5 after symptom onset, she arrived at the British Columbia Children’s Hospital (BCCH) emergency department with worsening headaches waking her from sleep. She was afebrile and had an unremarkable neurologic examination with no focal neurologic deficits, but her headache intensified throughout the day, and she was admitted to the hospital for further management.

Head computed tomography (CT) without contrast, CT venogram, and brain magnetic resonance imaging (MRI) were performed to assess for space-occupying lesions or sinus venous thrombosis. Results from those scans were unremarkable. On day 6 after symptom onset, clinicians performed a lumbar puncture (LP), which revealed an elevated opening pressure at 33.4 cm H_2_O (reference range 11.5–25 cm H_2_O). During LP, clinicians also collected a cerebrospinal fluid (CSF) sample. CSF analysis revealed an elevated nucleated cell count (NCC) of 250 × 10^6^ cells/L (reference range <10 × 10^6^ cells/L) (Table). She was treated empirically with ceftriaxone, vancomycin, and acyclovir after the LP.

**Table:** Clinical and demographic characteristics of patients in cluster of pediatric meningoencephalitis cases caused by snowshoe hare virus, Whistler, British Columbia, Canada, 2024*

Characteristics	Case 1	Case 2	Case 3
Patient age, y/sex	12/F	3/F	2/M
Clinical manifestations	5-d history of headaches, neck stiffness, photophobia, and emesis; afebrile	4-d history of fever and decreased energy; multiple hemiclonic focal seizures and subsequent Todd’s paresis on day 4 of illness	5-d history of fever, lethargy, and vomiting; bradycardia at admission
Medical history	No underlying conditions	History of mild hypoxic ischemic encephalopathy and previous seizure	Premature birth at 29 weeks’ gestation
Acute CSGV serology	8 d after symptom onset	5 d after symptom onset	13 d after symptom onset
IgM immunoassay			
SSHV	Reactive	Reactive	Reactive
JCV	Reactive	Nonreactive	Reactive
PRNT			
SSHV	1:1,280	1:80	1:2,560
JCV	1:640	1:40	1:640
Convalescent CSGV serology	6 wks after acute serology	4 wks after acute serology	2 wks after acute serology
IgM immunoassay			
SSHV	Reactive	Reactive	Reactive
JCV	Reactive	Reactive	Reactive
PRNT			
SSHV	1:640	1:2,560	1:1,280
JCV	1:320	1:640	1:640
CSF testing†	6 d after symptom onset	11 d after symptom onset	6 d after symptom onset
NCC, × 10^6^ cells/L (% lymphocytes)	250 (74)	12 (74)	112 (38); 51% neutrophils‡
Glucose, mmol/L	4.0	3.0	3.6
Protein, g/L	0.66	0.31	0.36
Head and brain imaging			
CT	Unremarkable	ND	Unremarkable
MRI	Unremarkable	Abnormal signal in the right perirolandic cortex, right insular cortex and bilateral thalami, with scattered areas of sulcal enhancement	ND


*CSF, cerebrospinal fluid; CSGV, California serogroup virus; CT, computed tomography; JCF, Jamestown Canyon virus; MRI, magnetic resonance imaging; NCC, nucleated cell count; ND, not done; PRNT, plaque reduction neutralization test; SSHV, snowshoe hare virus. †Reference ranges: NCC <10 × 10^6^ cells/L; glucose 2.8–4.4 mmol/L; protein 0.14–0.42 g/L. ‡Reference <6%.

CSF and blood cultures were negative, as was a BioFire Meningitis/Encephalitis CSF Panel (bioMérieux, https://www.biomerieux.com). CSF nucleic acid amplification test (NAAT) for Epstein-Barr virus (EBV) was also negative. Serologic tests for *Treponema pallidum*, Lyme disease, *Bartonella henselae*, *Toxoplasma gondii*, and West Nile virus (WNV) were nonreactive. EBV serology was IgM reactive but IgG nonreactive. Acyclovir and vancomycin were subsequently discontinued after 48 hours.

The patient continued to show clinical improvement and was discharged home after completing a 10-day course of ceftriaxone for culture-negative meningitis. On the day of discharge, she was doing well with only mild residual headaches. Repeat serology 6 weeks after initial serology was nonreactive for EBV IgM and IgG, suggesting a false-positive initial IgM result. 

We performed retrospective testing for CSGV, and those results were most consistent with SSHV infection on the basis of higher SSHV than JCV neutralizing antibody titers, although not a >4-fold increase in titers (Table). The result of RT-PCR for CSGV was negative on serum collected on day 8 after symptom onset, but insufficient residual CSF was available to perform further CSGV RT-PCR. Those serology findings supported a diagnosis of probable SSHV meningoencephalitis. The patient was seen for follow-up 3 months after discharge, and her symptoms continued to improve.

### Case 2

In early August 2024, a 3-year-old girl with history of strabismus and mild hypoxic ischemic encephalopathy with a single focal seizure 1 year earlier was admitted to the emergency department with 3 hemiclonic focal seizures and subsequent Todd’s paresis after a 4-day history of fever and decreased energy. She was treated with seizure medications and transferred to BCCH for further management.

At admission to BCCH, she was empirically treated with acyclovir. Two EEGs had abnormal findings; the first noted a focal electroclinical seizure with onset from the right posterior quadrant corresponding with left hand twitching, and the second showed posterior quadrant slowing. She defervesced by day 3 of admission and had 1 additional seizure on day 7 of hospitalization. Over the next few days, her parents reported that she was having visual hallucinations, such as seeing rainbows and seeing the room filled with water, as well as balance and speech difficulties.

Clinicians performed an LP on day 11 after symptom onset, and CSF analysis revealed a slightly elevated NCC at 12 × 10^6^ cells/L (Table). An MRI of the brain showed abnormal signal in the right perirolandic cortex, right insular cortex, and bilateral thalami, with scattered areas of sulcal enhancement. Results of CSF and blood cultures were negative. Results of BioFire Meningitis/Encephalitis CSF Panel performed on CSF samples and BioFire Respiratory 2.1 Panel (both bioMérieux) performed on nasopharyngeal swab samples were both negative. CSF 16S rRNA gene PCR result was also negative. Serologic tests for Lyme disease, cytomegalovirus, EBV, *Toxoplasma gondii*, *Bartonella henselae*, WNV, *Leptospira* spp., and *Rickettsia rickettsii* were nonreactive. Results of herpes simplex virus PCR of CSF samples, cryptococcal antigen tests of CSF and serum samples, and *Leptospira* blood and urine NAAT tests were also negative.

Acyclovir was subsequently discontinued after the negative herpes virus PCR. The patient remained hospitalized for 12 days and was subsequently discharged to home with regular seizure medications after clinical improvement but with lingering balance and speech issues. The patient was seen for follow-up 3 months after discharge with a resolution of symptoms and no further seizures.

We performed retrospective testing for CSGV using acute and convalescent serologies, which demonstrated a 32-fold rise in SSHV PRNT titers (Table), meeting the criteria for SSHV infection. RT-PCR for CSGV was negative on serum collected on day 5 of symptoms. Insufficient residual CSF was available to perform CSGV RT-PCR testing. 

### Case 3

In August 2024, a 2-year-old boy with a history of premature birth at 29 weeks’ gestation was taken to his local emergency department with a 5-day history of fever, lethargy, and vomiting. On examination, he was febrile, lethargic, and bradycardic. Clinicians administered an intravenous fluid bolus, obtained blood cultures, and treated him empirically with ceftriaxone and acyclovir, then transferred him to BCCH for further management.

A CT head scan at BCCH showed unremarkable results, and an LP on day 6 of symptom onset demonstrated an elevated NCC of 112 × 10^6^ cells/L (Table). Cultures of blood and CSF were negative. BioFire Meningitis/Encephalitis CSF Panel performed on CSF samples, BioFire Respiratory 2.1 Panel performed on nasopharyngeal swab samples, and BioFire Gastrointestinal Panel of stool samples (all bioMérieux) all had negative results. Serum cryptococcal antigen, *Leptospira* blood and urine NAAT, WNV serology, and *Rickettsia rickettsii* serologic test were negative. CSF 16S rRNA gene PCR result was also negative. An ECG and an echocardiogram showed borderline corrected QT prolongation and no abnormalities in cardiac anatomy*.*

The patient returned to his baseline and was defervescent by day 3 of admission. Acyclovir was discontinued within 48 hours, and he completed a 10-day course of ceftriaxone for culture-negative meningitis before he was discharged home. The patient was seen for follow-up 3 months later and was doing well.

We performed retrospective testing for CSGV using acute and convalescent serologies (Table). Those findings were most consistent with SSHV infection given the higher SSHV neutralizing antibody titers compared with JCV. Although we observed only a 2-fold change in SSHV titers between the acute and convalescent samples, that result could be because the acute CSGV serology was obtained 13 days after symptom onset. Furthermore, JCV titers did not change between acute and convalescent phases. Insufficient residual CSF samples were available to perform CSGV RT-PCR. 

## Public Health Investigation

Informed of the 3 cases, public health authorities launched a public health investigation. Whistler is a mountain resort municipality in British Columbia, known for extensive year-round outdoor activities, and has millions of visitors annually. All 3 case-patients spent time outdoors and were frequently exposed to wooded areas during hikes, camping activities, and evening outdoor activities. All case-patients had experienced a mosquito bite. Parents reported multiple possible exposure locations, but only 1 neighborhood in Whistler was common to all 3 cases during the exposure windows. That information supported targeted vector surveillance during the subsequent mosquito season.

In response to the cluster, regional public health released an update asking clinicians to report cases of encephalitis for which no pathogen was identified and that occurred during the 2024 mosquito season. That update resulted in identification of an additional adult case in a person who also resided in Whistler and initially had autoimmune encephalitis diagnosed. A residual banked CSF sample and acute serum sample and a convalescent serum sample collected in collaboration with the patient’s physician confirmed SSHV infection. That patient’s treatment for the presumed autoimmune encephalitis was discontinued. 

Furthermore, regional public health initiated an epidemiologic and environmental investigation during the 2025 mosquito season. That investigation included a small-scale mosquito surveillance pilot project in the same area to characterize local mosquito species and test them for potential pathogens like SSHV (*7*).

## Discussion

We describe a cluster of 3 cases of confirmed and probable pediatric SSHV meningoencephalitis from Whistler, British Columbia, Canada, during July–August 2024. All 3 cases had serologic evidence of recent CSGV infection most consistent with SSHV. All had a median symptom duration of 5 (range 4–5) days before hospital admission. None required intensive care unit admission, and all recovered fully. 

A previously reported cluster occurred in Quebec, Canada, in 1978, and involved 2 brothers with SSHV infection (*8*). In our cluster, diagnostic workup for CSGV was initiated only after initial investigations for meningoencephalitis were nondiagnostic and the treating clinicians recognized a possible epidemiologic link between the cases, spurring additional laboratory and public health investigations.

Our case series also highlights the variabilities in signs and symptoms and CSF findings in SSHV disease. Although pediatric viral meningoencephalitis typically includes lymphocytic predominance in CSF and variable protein and glucose levels, our small case series showed some variation. Two patients demonstrated lymphocytic predominance, but 1 had a slight neutrophilic predominance (Table). All 3 had CSF glucose levels within the reference range and protein levels within the reference range or slightly elevated. That variability is consistent with previous case reports of CSGV meningoencephalitis, which have also noted neutrophilic pleocytosis in CSF (*9*). Those results could be related to the timing of CSF sample collection relative to symptom onset because CSF samples obtained early in viral meningoencephalitis can exhibit a neutrophilic predominance.

The 3 cases in this study further highlight the complexity of diagnosing CSGV. None of the patients were positive for CSGV by RT-PCR from serum, reflecting known lower RT-PCR sensitivity. Exhaustive testing for the initial infectious disease workup left no residual CSF samples for retrospective RT-PCR, highlighting the need for an early high index of clinical suspicion. However, 1 patient met the diagnostic criteria for confirmed CSGV infection on the basis of serologic testing, and the other 2 were probable cases with a strong epidemiologic link (Appendix). 

Using paired serum samples typically requires a >4-fold increase in neutralizing antibodies to the specific virus PRNT titers in the convalescent serum sample to confirm acute infection because 2-fold changes can reflect assay variability (*5*). Detection of CSGV IgM by ELISA in a single serum sample is insufficient for diagnosis because those results might reflect prior infection or cross-reactivity within CSGVs. The high CSGV seroprevalence in Canada, estimated at up to 20%–30% depending on the region, further complicates interpretation of a single serum result because persistence of IgM for several months after infection has been described for both SSHV and JCV (*4*,*10*–*12*).

All 3 patients exhibited detectable IgM against both SSHV and JCV by ELISA. However, paired acute and convalescent PRNT titers indicated SSHV as the most likely causative agent. Case-patient 1 had a 2-fold decrease in SSHV PRNT titers over 6 weeks, but those titers were consistently higher than JCV titers, which also decreased by 2-fold. Case-patient 2 had a 32-fold rise in SSHV PRNT titers over 4 weeks, meeting the case definition for SSHV infection. Case-patient 3 had a 2-fold decrease in SSHV PRNT titers over 2 weeks and no change in JCV titers; the relatively higher initial SSHV titers compared with static JCV titers supported SSHV as the more likely virus. Of note, 2 of the patients had decreases in SSHV neutralizing antibody titers in convalescent serum samples compared with acute serum samples, which is opposite of expected results. However, patient 1 had initial serology collected at day 8 after symptom onset and patient 3 had initial serology collected at day 13 after symptom onset, in contrast to patient 2, who had initial serology collected at day 5 after symptom onset. The relatively delayed acute serum sample collection for patients 1 and 3 demonstrated higher initial SSHV titers than that of patient 2, likely reflecting a more established serologic response that would be expected later in the infection. Those findings highlight the importance of paired acute and convalescent serologic testing at the appropriate time interval and PRNT confirmation in diagnosing SSHV/CSGV infections. However, the strong epidemiologic link between the 3 cases strengthens the SSHV diagnosis in the 2 patients who might not have met the definition requiring a >4-fold rise in neutralizing antibodies from acute versus convalescent titers.

Few reports of neuroinvasive disease caused by SSHV are in the literature, even though seroprevalence studies in animals and humans would suggest relatively high rates of exposure in at least some geographic regions of North America, where positive CSGV serology ranges from 18% to 75% (*10*–*12*). For example, a serologic survey of snowshoe hares in the greater Yellowstone National Park area during 2009–2012 found SSHV seroprevalence rates ranging from 18% to 48% depending on the year, mirroring previous findings from Canada and Alaska in the 1960s that reported CSGV seroprevalence of 20%–75% in snowshoe hares (*13*). A 2015–2016 human serosurvey in New Brunswick, Canada, revealed that 31% of serum samples had CSGV antibodies, and further PRNT testing indicated that 26% of single-virus exposures were JCV and only 2% were SSHV (*14*). However, serology might not be able to definitively distinguish between those 2 CSGVs (*15*). Furthermore, 49.8% of encephalitis-associated cases in Canada have no diagnosed cause (*9*). Taken together, those findings suggest that SSHV-associated neuroinvasive disease is likely underdiagnosed and underreported in North America. Thus, identification of 4 confirmed neuroinvasive cases of SSHV infection in such a short timeframe and small geographic region has substantial clinical and public health implications.

One hypothesis for this cluster is that a higher than usual SSHV prevalence in the mosquito population that season led to a relatively high rate of exposure in the human population, resulting in detection of more neuroinvasive disease. Climate change could affect the distribution of CSGV hosts and, more crucially, the arthropod vectors (*16*). Limited empiric evidence has shown that warming temperatures can increase the winter survival of viruses in mosquitoes and, hence, the rate of transovarial transmission of viruses within those mosquitoes (*17*,*18*). SSHV infectivity also has been shown to be higher in mosquitoes incubated at higher temperatures (*11*). Furthermore, SSHV continues to be isolated from commonly captured mosquito species in Canada, typically *Aedes* spp., which are abundant and widespread (*10*,*14*). Those factors could lead to increased SSHV transmission in endemic regions and a broader distribution of the virus and its vectors.

Of note, >200 human cases of SSHV and JCV infection have been documented across Canada since 2006, and ≈70% of those were confirmed as JCV infections (*2*). However, our cluster of cases might indicate a shifting trend toward a higher SSHV prevalence in some parts of the country. That shift might reflect changes in virus circulation or vector or host distribution or could be attributed to improved serologic testing. That shift also potentially explains why our case series was among pediatric patients because most symptomatic SSHV infections have been identified in children, but symptomatic JCV infections have been more frequently identified in adults (*2*,*10*,*19*). Future public health surveillance of mosquitoes for CSGV as well as updated seroprevalence studies could help substantiate that hypothesis. More studies are needed to delineate the true incidence of SSHV infections in North America.

## Conclusions

This case series suggests an underrecognized incidence of SSHV infection that warrants enhanced future surveillance and review of existing diagnostic algorithms. In addition, many jurisdictions have no active mosquito surveillance, and the cases we describe highlight the need to reinvest in such surveillance to clarify risks for SSHV infection among humans. Clinicians in North America should consider CSGV, including SSHV, as a potential cause of neuroinvasive disease during mosquito season, particularly when initial diagnostic testing is inconclusive. 

Appendix Additional information on pediatric meningoencephalitis cases caused by snowshoe hare virus, Whistler, British Columbia, Canada, 2024.
