# Supplementary material for: Pediatric Meningoencephalitis Cluster Caused by Snowshoe Hare Virus, Whistler, British Columbia, Canada, 2024
Source: Emerg Infect Dis. 2026 Apr;32(4):477–83. doi: 10.3201/eid3204.251392 (PMC13094835; doi:10.3201/eid3204.251392)
Supplement: Appendix — Additional information on pediatric meningoencephalitis cases caused by snowshoe hare virus, Whistler, British Columbia, Canada, 2024. [file 25-1392-Techapp-s1.pdf]

Article DOI: https://doi.org/10.3201/eid3204.251392

EID cannot ensure accessibility for supplementary materials supplied by authors. Readers who have difficulty accessing supplementary content should contact the authors for assistance.

Pediatric Meningoencephalitis Cluster Caused by Snowshoe Hare Virus, Whistler, British Columbia, Canada, 2024

Appendix

Snowshoe Hare Virus (SSHV) Laboratory Diagnosis

IgM Antibodies

IgM specific to SSHV are detected using enzyme-linked immunosorbent assays (ELISA). These can be done on cerebrospinal fluid (CSF) or serum. The presence of IgM typically indicates recent exposure to SSHV. There is a large degree of cross reactivity between the California serogroup viruses (CSGV). Furthermore, IgM antibodies can persist for up to one to 2 years in some arboviruses. Therefore, confirmatory testing such as plaque reduction neutralization testing (PRNT) is required to confirm the diagnosis. Furthermore, high seroprevalence to CSGV in North America makes it difficult to differentiate recent from past infections from a single serum specimen, thus the need for paired serum and/or CSF (*1*).

Plaque Reduction Neutralization Test (PRNT)

PRNT is the standard for confirming SSHV infection. It measures the ability of serum to neutralize the virus, preventing plaque formation and can sometimes differentiate between the different viruses within the CSG (*2*).

PCR

PCR on blood or CSF detects SSHV genetic material with high specificity, useful for early diagnosis before seroconversion has occurred. However, it is typically only positive within the first few days of symptom onset and is not routinely recommended for immunocompetent individuals given its lower sensitivity (*3*).

In our cases, serum samples submitted to the NML were first analyzed by SSHV and JCV IgM ELISA to determine if the patients had developed antibodies to SSHV and/or JCV. Both acute and convalescent serologies (range: 2 to 6 weeks apart) were done. As all samples were SSHV IgM positive, they were further analyzed via PRNT for the presence of neutralizing antibodies to SSHV and/or JCV.

Snowshoe Hare Virus (SSHV) Infection Case Definitions

Confirmed case: Defined as a 4-fold or greater change in virus-specific antibody titer, presence of either virus-specific IgM or neutralization antibodies in cerebrospinal fluid, or detection of virus-specific RNA in cerebrospinal fluid or isolation of virus by cell culture (*1*,*2*,*4*).

Probable case: Defined as an individual with a clinically compatible illness (with symptoms observed during the mosquito season) and detectable SSHV IgM antibody and virus specific neutralization antibodies in the acute serum sample (*1*,*2*,*4*).

References

1. Canadian Network for Public Health Intelligence. Detection of IGM antibodies directed towards snowshoe hare virus by ELISA [cited 2025 Sep 12]. https://cnphi.canada.ca/gts/reference-diagnostic-test/5384

2. Canadian Network for Public Health Intelligence. Detection of antibodies directed towards snowshoe hare virus by PRNT [cited 2025 Sep 12]. https://cnphi.canada.ca/gts/reference-diagnostic-test/5385

3. Canadian Network for Public Health Intelligence. Molecular Detection of snowshoe hare virus by Reverse Transcriptase PCR (RT-PCR) - Guide to Services [cited 2025 Sep 12]. https://cnphi.canada.ca/gts/reference-diagnostic-test/5383

4. Drebot MA. Emerging mosquito-borne bunyaviruses in Canada. Can Commun Dis Rep. 2015;41:117–23. PubMed https://doi.org/10.14745/ccdr.v41i06a01
